# Characterization of off-odours and potentially harmful substances in a fancy dress accessory handbag for children

**DOI:** 10.1038/s41598-017-01720-5

**Published:** 2017-05-11

**Authors:** Christoph Wiedmer, Cristina Velasco-Schön, Andrea Buettner

**Affiliations:** 1 Friedrich-Alexander-Universität Erlangen-Nürnberg, Professorship for Aroma Research, Emil Fischer Center, Department of Chemistry and Pharmacy, Henkestrasse 9, 91054 Erlangen, Germany; 2 Department Sensory Analytics, Fraunhofer Institute for Process Engineering and Packaging IVV, Giggenhauser Straße 35, 85354 Freising, Germany; 3 Bayerisches Landesamt für Gesundheit und Lebensmittelsicherheit, Sachgebiet Bedarfsgegenstände (Bavarian Health and Food Safety Authority, Department of articles of daily use), Eggenreuther Weg 43, 91058 Erlangen, Germany

## Abstract

A fancy dress accessory handbag for children was claimed by consumers to exhibit an offensive smell. Sensory characterization by an expert panel revealed, amongst others, rubber- and car tire - like notes. For elucidation of the molecular reasons of this sensory defect, the volatile fraction of the product was isolated by means of solvent extraction and high vacuum distillation. Identification of the main odorants was accomplished by means of one- and two-dimensional gas chromatography, with parallel mass spectrometric and olfactometric detection. In total more than 60 odorants were detected in the sample and more than 30 of these odour-active substances could be identified. Amongst them were a number of naphthalene derivatives as well as saturated and mono- or di-unsaturated carbonyl compounds. The naphthalene derivatives that were identified in the children’s article appeared to be mainly responsible for the characteristic off-odour. Additionally, a GC-MS-screening for polycyclic aromatic hydrocarbons (PAHs) was performed, which revealed the presence of 15 PAHs in total. However, 14 of them were of no relevance for the smell of the product.

## Introduction

The sales of toys in Germany have steadily increased over the last decade^1^. With the increasing variance of toys, the range of materials that are used for their manufacture has likewise expanded. To ensure that only safe products are provided on the market, a variety of European and national laws and regulations set standards for the safety of toys. Nevertheless, the Rapid Alert System for dangerous non-food products of the European Commission shows that hundreds of unsafe toys have to be withdrawn from the market every year^2^. Considering the 468 toys that were added to this database in the year 2015, choking hazards and chemical risks were the main reasons for a recall of 196 and 195 affected products, respectively^2^. It is also worth mentioning that 88.2% of the toys claimed to be dangerous in 2015 stemmed from China^2^.

Since hazardous chemicals are an important issue in the safety of toys, it is understandable that German food safety authorities receive several complaints about malodorous toys by worried customers each year. As reported by German media, there are also several examples where malodorous toys contained dangerous substances so that the product had to be recalled; one example is an ambulance car toy that contained high amounts of benzo(a)pyrene^3^. Another example is an inflatable radio controlled minion toy, which was recently recalled after high amounts of naphthalene were found in this product^4^. Furthermore, malodorous toys need to be regarded with caution due to the fact that the smell of toys can influence a child’s behaviour in a significant way^5^.

However, the smell of toys is not specifically regulated by German or European law, except for the application of fragrance allergens. Nevertheless, the German Federal Institute for Risk Assessment (Bundesinstitut für Risikobewertung, BfR) advises consumers to avoid intensively smelling products^6^. In general, smells relating to material emissions have hitherto been rarely investigated on a molecular basis and little is known about the exposure of customers to such substances. Accordingly, published data on odorous contaminants in toys and articles of daily use are rare.

In our research group, three scientific theses were previously conducted aiming at the identification of odorants in toys and articles of daily use; these studies indicate that a variety of odour-active compounds can be found in such products. In the study by Husko (2012) a selection of 20 toys and articles of daily use was evaluated by a sensory panel^7^. Based on this evaluation, four products (a hairbrush, an inflatable pillow, a beach ball and balloons) were found to exhibit a noticeable smell^7^. These samples were then applied to further testing by means of gas chromatography-olfactometry (GC-O) and two-dimensional gas chromatography-olfactometry/mass spectrometry (2D-GC-MS/O)^7^. Using this approach, substances are not only recorded by means of analytical detectors but are additionally evaluated by human assessors. Therefore, this specialized procedure ensures that odorous substances can be specifically targeted even amongst a large number of non-odorous volatiles.

Husko could show that the balloons contained the coconut-like smelling substances δ-nonalactone, γ-nonalactone, γ-decalactone, and the faecal smelling indole^7^. The floral smelling β-ionone, the sweet smelling 4-anisaldehyde, the sweet/floral smelling coumarin and the coconut-like smelling substances δ-nonalactone, γ-nonalactone and δ-decalactone were identified in the inflatable pillow^7^. In contrast to that, 2,3-diethyl-5-methylpyrazine (musty/earthy smell), 4-phenylcyclohex-1-en (carpet-like smell), p-cresol (horse stable-like smell), 4-ethylphenol (horse stable-like, ink-like smell), 3-isopropylphenol (leather-like, phenolic smell), 4-isopropylphenol (rubber-like, phenolic smell) and 2,4,5-trimethylphenol (leather-like, phenolic smell) were found in the beach ball^7^; accordingly, the smell of this product was dominated by aromatic, phenolic compounds. The hairbrush contained 4-ethylphenol, 2,4,5-trimethylphenol, p-cresol, 3-isopropylphenol and 4-phenyl-1-cyclohexene, which were also identified in the beach ball, γ-nonalactone, 4-anisaldehyde and β-ionone, which were also found in the inflatable pillow, and oct-1-en-3-one (mushroom-like smell) and 3-methylindole (faecal smell)^7^.

Odorants that caused a strong off-odour in a plastic pony were analyzed by Kröner^8^. The article contained the cheesy smelling butanoic acid, the mushroom-like smelling oct-1-en-3-one, the phenolic smelling phenol, the savoury-like smelling sotolone, the honey-like smelling phenylacetic acid, the metallic smelling trans-4,5-epoxy-(*E*)-dec-2-enal, the vanilla-like smelling vanillin, the fishy/fatty smelling dodecanoic acid and the peach-like smelling γ-dodecalactone. Moreover, the fatty/tallowy/vegetable-like smelling substances (*E*,*Z*)-nona-2,6-dienal, (*E*)-non-2-enal, (*E,E*)-nona-2,4-dienal and (*E*,*E*)-deca-2,4-dienal were identified as additional potent odorants.

In a recent study, sensory evaluation of 25 plastic products was performed by Leichsenring^9^. On the basis of the intensity ratings by the sensory panel and the estimated consumers’ skin contact four products (rubber bands, plastic clogs, a night light and a plastic ball) were selected for the identification of odorants in these products^9^. In total, 27 odorants were successfully identified^9^. Twelve of these odorants were found in at least three of the four samples; these were the fatty smelling substances (*E*,*E*)-deca-2,4-dienal, (*E*)-non-2-enal, (*Z*)-non-2-enal, substances with a mushroom-like smell (non-1-en-3-one and oct-1-en-3-one) and the citrus-like smelling substances nonanal and octanal^9^. Furthermore trans-4,5-epoxy-(*E*)-dec-2-enal (metallic), linalool (sweet, floral), skatole (faecal), 2,2,4-trimethyl-1,3-pentandiol diisobutanoate (plastic-like) and vanillin (vanilla-like) were identified^9^.

Apart from the context of toys, there are also publications addressing the occurrence and formation of odorants in plastics. Bravo *et al*.^10^ exposed polypropylene to high temperatures (250 °C) for 15 minutes and analyzed the volatile compounds escaping the polymer by means of GC-O^10^. Several aldehydes like hexanal, nonanal or (*E*)-non-2-enal and ketones like diacetyl, hept-1-en-3-one, oct-1-en-3-one or non-1-en-3-one could be identified as thermal oxidation products of polyethylene^10^.

A study by Morrison and Nazaroff (2002) demonstrates that the presence of ozone can also lead to the formation of odour-active compounds in plastics^11^. For their research carpets made from nylon or olefin fibre were exposed to 100 ppb ozone and the emission of volatiles from the carpets was measured using GC-MS^11^. All exposed samples emitted, amongst other odorants, nonanal, and one sample also emitted high amounts of an unspecified isomer of 2-nonenal^11^. However, the compounds generated were not rated using olfactometric techniques. Accordingly, it remains unclear if potent odour-active substances escaped detection.

Mayer and Breuer (2006) proposed that another pathway to be considered for the formation of unsaturated aldehydes is the autoxidation of unsaturated fatty acids^12^. According to the authors, unsaturated fatty acids in plastics can stem from fatty lubricants used during production of such products^12^.

Accordingly, only little is known about odorants in plastic matrices. To the best of our knowledge, apart from the aforementioned student theses, there is no data available on odorants in children’s products or toys. Likewise, peer-reviewed publications addressing the chemical composition of toys are rare. However, these few previous studies indicate that a variety of substances and chemical substance classes may be responsible for off-odours in toys. As such, the aim of our study was to extend the knowledge of odorous contaminants and odourless hazardous chemicals in toys by exemplarily analysing a handbag that was sold as an accessory for a children’s costume and that was reported as offensively smelling. Therefore, an expert sensory analysis was employed as well as one- and two-dimensional gas chromatography experiments with olfactometic and mass spectrometric detection, including a flavour dilution approach. Additionally, a screening analysis for polycyclic aromatic hydrocarbons (PAHs) was performed.

## Results

### Sensory evaluation

The smell of the sample was rated as very intense with a value of 8.2. In the course of the descriptive analysis the following odour attributes were chosen by the panellists: sweet, phenolic, artificial leather-like, plastic-like, car tire-/rubber-like, wood-like, diesel-/gasoline-like, mothball-like, cheesy/sweaty, vinegar-like and pungent. Apart from that, panellists reported a burning sensation in the eyes or the nose and a pungency sensation that was rated according to the same scale as the other odour attributes.

**Figure 1:**
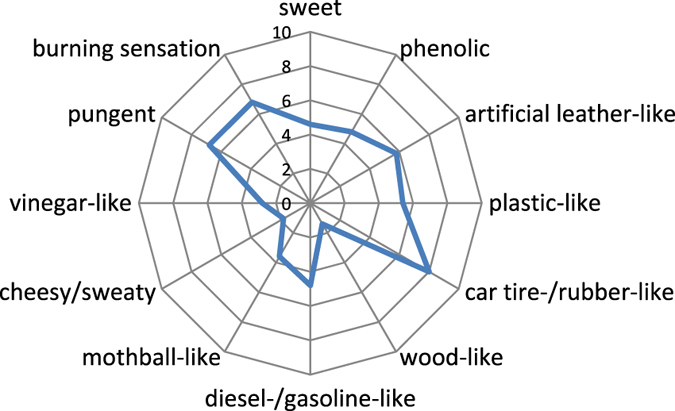
Odour profile of the investigated sample. The data are displayed as mean numerical values of the sensory evaluation of five panellists.

According to the results of the odour profile analysis, the *car tire*- or *rubber-like* smell was the most dominant impression with a rating of 8.0. The attributes *pungent* and *burning* were both rated with 6.8 relating to an intense perception. Values of 5.8 and 5.4, respectively, show that there was also a strong perception of *artificial leather-like* and *plastic-like* notes. The attributes *diesel-*/*gasoline-like*, *phenolic*, *sweet* and *mothball-like* were rated with values of 4.8, 4.8, 4.6, and 3.6, and were, accordingly, clearly perceivable. With mean ratings of 2.8, 1.8 and 1.4, vinegar-like, cheesy/sweaty and wood-like notes were perceived with weak intensities. The odour profile of the sample is shown in Fig. 1.

### GC-O analysis of the sample

GC-O analysis showed that 62 odorants could still be detected in the diluted extract corresponding to flavour dilution (FD) factor 9 when evaluating the sample on capillary DB-FFAP. Olfactometric evaluation of the undiluted extract and of the diluted extract corresponding to FD3 was not performed due to the overwhelming nature of the smell constituents in these samples, and due to work safety considerations. On the other hand, eleven of these 62 odorants were still detectable in the highest dilution step FD 729. In the following, the identification work was focused on the most potent odorants and odorants with unpleasant smells in the FD range from 9 to 729.

With high FD-factors, several fatty smelling substances were found, such as (*E*)-non-2-enal, (*E*,*E*)-deca-2,4-dienal, (*E*,*E*)-nona-2,4-dienal and (*E*,*Z*)-nona-2,4-dienal. Additionally, the naphthalene-like smelling substances 1,2-dimethylnaphthalene, 1-methylnaphthalene, 2-methylnaphthalene and naphthalene could be identified. Other odorants with high FD-factors identified by GC-O were 3-methylisoquinoline (sweet, portable toilet-like), raspberry ketone (sweet, berry-like), rotundone (pepper-like), sotolone (spicy), trans-4,5-epoxy-(*E*)-dec-2-enal (sweet, metallic) and vanillin (vanilla-like).

### GC-MS/O analysis of the sample

**Figure 2:**
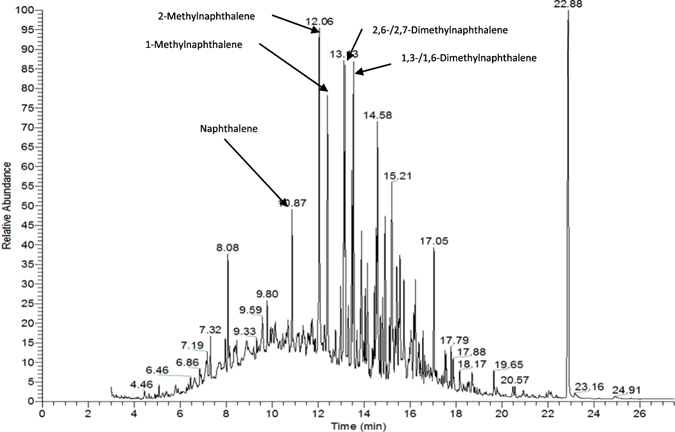
GC-MS-chromatogram of the diluted sample extract corresponding to FD 9. The largest peaks identified as naphthalene derivatives are indicated with arrows.

After one-dimensional gas chromatographic separation of the diluted sample extract corresponding to FD 9, mass spectra of naphthalene and several methyl- and dimethylnaphthalenes were successfully obtained and aligned with the spectra of the corresponding reference substances. The identified compounds, namely naphthalene, 1-methylnaphthalene, 2-methylnaphthalene, 1,2-dimethylnaphthalene, 1,4-dimethylnaphthalene, 1,5-dimethylnaphthalene, 1,6-dimethylnaphthalene, 1,7-dimethylnaphthalene and 2,6/2,7-dimethylnaphthalene, are listed in Table 1. Commonly, the naphthalene derivatives exhibited characteristic moldy, leather- and rubber-like smells, which were described as naphthalene-like. However, 2,6- and 2,7-dimethylnaphthalene smell anise-like. As shown in Fig. 2, the majority of the peaks corresponding to the identified naphthalene derivatives were amongst the highest peaks in the chromatogram.

**Table 1:** List of all identified odorants in the sample by means of GC-O and their characteristics.

No.	Odorant	Odour quality^a^	Ri value^b^ on		FD-factor^c^ on		Identified by
			DB-FFAP	DB-5	DB-FFAP	DB-5	
1	(*E*)-Non-2-enal	fatty	1521	1156	≥729	81	d
2	(*E*)-Oct-2-enal	fatty, peanut-like	—	1058	—	27	i
3	(*E,E*)-Deca-2,4-dienal	fatty, peanut-like	1808	—	≥729	—	i
4	(*E,E*)-Nona-2,4-dienal	fatty, peanut-like	1686	1212	≥729	≥729	d
5	(*E,E*)-Nona-2,6-dienal	cucumber-like, green	—	1153	—	81	i
6	(*E,E*)-Octa-2,4-dienal	cucumber-like	1579	1100	81	81	h
7	(*E,Z*)-Nona-2,4-dienal	fatty, cucumber-like	1657	1189	243	27	h
8	(*Z*)-Non-2-enal	green	1493	—	27	—	i
9	1,2-Dimethylnaphthalene	naphthalene-like	2042	1453	≥729	243	d, e
10	1,4-Dimethylnaphthalene	naphthalene-like	—	1457	n.d.	n.d.	g
11	1,5-Dimethylnaphthalene	naphthalene-like	2017	—	n.d.	n.d.	f
12	1,6-Dimethylnaphthalene	naphthalene-like	—	1440	n.d.	n.d.	g
13	1,7-Dimethylnaphthalene	naphthalene-like	1978	—	n.d.	n.d.	f
14	1-Methylnaphthalene	naphthalene-like	1869	1306	≥729	27	e
15	2,3,5-Trimethylnaphthalene	naphthalene-like, leather-like	2155	—	9	—	d
16	2,6-/2,7-Dimethylnaphthalene	anise-like	1948	1420	n.d.	n.d.	f, g
17	2-/3-Methylbutanoic acid	cheesy	—	863	—	9	i
18	2-Methylnaphthalene	naphthalene-like	1831	1294	≥729	243	e
19	3-Ethylphenol	phenolic, leather-like	2173	—	≥729	—	d
20	3-Methylisoquinoline	sweet, portable toilet-like	1975	1325	243	≥729	d
21	Acetophenone	almond-like, solvent-like	1643	1076	81	9	h
22	Benzothiazole	car tire-like	1917	1221	9	81	h
23	Butanoic acid	cheesy	1629	—	27	—	i
24	Dodecanoic acid	fatty, fishy	2470	1573	81	81	h
25	Hex-1-en-3-one	glue-like	—	<800	—	27	i
26	Hexanal	cut grass-like, tomato-like, green	1077	806	81	81	h
27	Naphthalene	mouldy, naphthalene-like	1719	1183	243	≥729	e
28	Nonanal	green, grassy	1381	1111	81	9	h
29	Oct-1-en-3-one	mushroom-like	1288	979	27	27	h
30	Octanal	cucumber-like, green	1276	1000	9	27	h
31	p-Cresol	horse stable-like	2075	1087	243	81	d
32	Phenylacetic acid	honey-like	2550	—	81	—	i
33	Raspberry ketone	sweet, berry-like	2973	1560	≥729	≥729	h
34	Rotundone	pepper-like	2245	1715	≥729	≥729	h
35	Sotolone	spicy	2191	1117	≥729	27	h
36	trans-4,5-Epoxy-(*E*)-dec-2-enal	sweet, metallic	1942	1381	81	≥729	d
37	Vanillin	vanilla-like	2560	1400	≥729	≥729	d
38	γ-Octalactone	sweet, coconut-like	—	1265	—	81	i

^a^Odour quality perceived at the sniffing port during GC-O analysis.

^b^Retention index on capillaries DB-FFAP and DB-5 according to^24^.

^c^FD: determined flavour-dilution factor according to^25^.

^d^The compound was identified by comparison of odour quality, retention index on both capillaries and mass spectrum (MS-EI), obtained by 2D-GC-MS/O analysis, with the properties of the reference compound.

^e^The compound was identified by comparison of odour quality, retention index on capillary DB-FFAP and mass spectrum (MS-EI), obtained by GC-MS/O analysis, with the properties of the reference compound.

^f^The compound was identified by comparison of retention index on capillary DB-FFAP and mass spectrum (MS-EI) only, obtained by GC-MS/O analysis, with the properties of the reference compound.

^g^The compound was identified by comparison of retention index on capillary DB-5 and mass spectrum (MS-EI) only, obtained by GC-MS/O analysis, with the properties of the reference compound.

^h^The compound was identified by comparison of odour quality and retention indices on capillaries DB-FFAP **and** DB-5.

^i^Proposed structure by comparison of odour quality and retention index on capillaries DB-FFAP **or** DB-5.

n.d. not determined.

Apart from naphthalene derivatives no other odorants could be identified using one-dimensional GC-MS/O since their mass spectra were covered by a range of odourless substances. To focus the MS analysis on these odorants, 2D-GC-MS/O was used in the next step.

### 2D-GC-MS/O analysis of the sample

Identification of the remaining odorants by means of 2D-GC-MS/O analyses focused in particular on those odorants showing unpleasant smells and/or high FD factors. Thereby, a series of compounds were identified; these were (*E*)-non-2-enal (fatty), (*E*,*E*)-nona-2,4-dienal (fatty, peanut-like), 2,3,5-trimethylnaphthalene (naphthalene-like, leather-like), 3-ethylphenol (phenolic, leather-like), 3-methylisoquinoline (sweet, portable toilet-like), p-cresol (horse stable-like), trans-4,5-epoxy-(*E*)-dec-2-enal (sweet, metallic) and vanillin (vanilla-like).

Further attempts were targeted at the separation and alignment of all dimethylnaphthalene isomers by means of 2D-GC-MS/O. However, for this task the used methods did not generate further information than already obtained by one-dimensional GC-MS/O due to restrictions in the chromatographic resolution of these compounds. Table 1 provides an overview of all substances identified in the sample.

### Screening analysis for PAHs

In addition to naphthalene, which was also identified by means of GC-O, the screening analysis for PAHs revealed the presence of 15 different PAHs in the sample, some with critically high concentrations. The substances with the highest concentrations measured were phenanthrene (8.28 mg/kg), flourene (4.91 mg/kg) and naphthalene (3.60 mg/kg). Table 2 provides an overview of all identified PAHs.

**Table 2:** Identified PAHs in the sample. Concentrations marked with a * did not lie within the calibration range and were therefore only estimated.

No.	Substance	Concentration [mg/kg]
1	Naphthalene	3.60
2	Acenaphthylene	1.32
3	Fluorene	4.91
4	Phenanthrene	8.28
5	Anthracene	2.82
6	Fluoranthene	0.98
7	Pyrene	2.07
8	Benzo[a]anthracene	0.48
9	Chrysene	1.36
10	Triphenylene	1.04*
11	Benzo[b]fluoranthene	0.25
12	Benzo[k]fluoranthene	0.25
13	Benzo[e]pyrene	0.47
14	Benzo[a]pyrene	0.10*
15	Benzo[g,h,i]perylene	0.04*

## Discussion

GC-O analyses led to the detection of more than 60 odorants; thereby, aroma extract dilution analysis (AEDA) revealed 22 substances to be amongst the most odour potent constituents. Of these, 12 substances were successfully identified using one- and two-dimensional GC-MS analyses.

Overall, the smell of the sample was comparable to the smell of the identified naphthalene derivatives. This also correlates with the fact that the peaks for the naphthalene derivatives were amongst the highest peaks in the chromatogram. Due to their predominantly comparable smell it is likely that naphthalene and its methyl and dimethyl derivatives show at least an additive if not even synergistic effect so that this substance group as a whole is perceived as much more intense than its single constituents. A comparison of the attributes that were named during sensory evaluation and the odour qualities perceived during GC-O indicates that the leather-like smell might be due to 3-ethylphenol whereas the car tire-like notes might result from the presence of benzothiazole.

Amongst the identified odorants were also several green or fatty smelling substances that were identified as fatty acid derived compounds with high FD-factors; these were: (*E*)-non-2-enal, (*E*,*E*)-deca-2,4-dienal, (*E*,*E*)-nona-2,4-dienal and (*E*,*Z*)-nona-2,4-dienal. The cucumber-like smelling substances (*E*,*E*)-nona-2,6-dienal and (*E*,*E*)-octa-2,4-dienal were also found, but with lower FD-factors. It is interesting to note, however, that fatty and green smells were not reported as attributes during the sensory evaluation. The reason might be that the other aforementioned substances, namely naphthalene and its derivatives, suppress the perception of the fatty smelling compounds due to their abundant nature.

Interestingly, there was a high rating of the “pungent” and “burning” sensations in the sensory evaluation. However, during GC-O analysis no specific substance was detected that specifically exerted such an effect that is commonly related with an activation of the TRP channels of the Nervus Trigeminus^13^. Accordingly, it is unclear if the olfactorily detected substances, and potentially even some of the odourless compounds, act as a whole to generate this trigeminal effect. Future studies comprising quantification and reconstitution experiments would be required to answer this question.

During GC-O analysis some smells could only be detected on one capillary, others showed high differences in FD-factors between the two capillaries. This can be explained by the fact that non-optimal interaction between the odorant and the stationary phase may lead to peak spreading and, consequently, lower concentrations of the odorants at the respective peak maxima so that these substances are perceived with lower intensities. Apart from that, the smell of odorants might also be covered by other co-eluting odorous substances.

Our study further shows that not every dimethylnaphthalene isomer that was found using GC-MS could be detected using GC-O. One reason might be that the different isomers have different odour thresholds. Another explanation may be that some isomers cover others by cross-adaptation, meaning that one naphthalene derivative that is smelled shortly before the next one may negatively impact the perception of the latter. But even though the smell of these substances could not be perceived using GC-O, they may show a synergistic effect with other naphthalene derivatives. It also needs to be mentioned that dimethylnaphthalenes have not been reported as off-odorants in plastics before, so there is no data available to verify these hypotheses.

Sources for the identified odorants seem to be as different as their chemical structures. PAHs such as naphthalene, for example, can be transferred into toys by contaminated extender oils or *Carbon black*, a pigment used in rubber and plastic products^14^. It seems to be likely that the methyl- and dimethylnaphthalenes found in the sample stem from the same sources. In view of this, it is important to note that the fancy dress accessory was coloured black. The (poly-)unsaturated aldehydes, on the other hand, are likely to be autoxidation products of fatty acids, as proposed by Mayer and Breuer^12^. Benzothiazole can be formed from 2-mercaptobenzothiazole or 2-morpholinodithiobenzothiazole; both substances are used in the vulcanization of rubber^15^.

It is also worth mentioning that several of the identified odorants from the handbag sample have been previously been reported in toys or other matrices. Table 3 provides an overview of such findings in other products.

**Table 3:** Examples for the occurrence of identified odorants in other products or matrices.

No.	Odorant	Previously identified in
1	(*E*)-Non-2-enal	toys^8, 9^, polypropylene powder^26^
2	(*E,E*)-Deca-2,4-dienal	toys^8, 9^
3	(*E,E*)-Nona-2,4-dienal	toys^8, 9^
4	(*Z*)-Non-2-enal	toys^9^, polypropylene powder^26^
5	1-Methylnaphthalene	flip-flops^27^, food (cooked lobster^28^, tonka beans^29^, bell peppers^30^)
6	2,3,5-Trimethylnaphthalene	scallops^31^
7	2-/3-Methylbutanoic acid	polypropylene powder^26^
8	2-Methylnaphthalene	flip-flops^27^, food (scallops^31^, bell peppers^30^)
9	Acetophenone	plastic shoes^6, 27^, toys^27^
10	Benzothiazole	a mattress^15^
11	Butanoic acid	toys^8, 9^, polypropylene powder^26^
12	Dodecanoic acid	toys^8, 9, 32^
13	Hexanal	polypropylene powder^26^, thermal oxidation product of polyethylene^10^, food (e.g. scallops^31^, tonka beans^29^, bell peppers^30^)
14	Naphthalene	articles of daily use (cables, flooring, a mouse-pad, flip-flops^27^, a mattress^15^), toys^14^; food (e.g. scallops^31^, smoked cheese^33^, bell peppers^30^)
15	Nonanal	toys^9^, polypropylene powder^26^, thermal oxidation product of polyethylene^10^, scallops^31^
16	Oct-1-en-3-one	articles of daily use^7^, toys^9^, thermal oxidation product of polyethylene^10^, polypropylene powder^26^
17	Octanal	toys^9^, polypropylene powder^26^, food (e.g. scallops^31^, tonka beans^29^)
18	Phenylacetic acid	toys^8, 9^, polypropylene powder^26^
19	Rotundone	toys^9^
20	trans-4,5-Epoxy-(*E*)-dec-2-enal	toys^8, 9^, polypropylene powder^26^
21	Vanillin	toys^8, 9^, polypropylene powder^26^

Some of the malodorous substances identified in the handbag need to be addressed with special concern since they might also be hazardous. Naphthalene, which was found in the sample, is a class 2 carcinogen (suspected human carcinogen) according to European Regulation (EC) No. 1272/2008 Annex VI part 3^16^. Data available for the identified substance 2-methylnaphthalene “are inadequate to assess human carcinogenic potential” according to the US Environmental Protection Agency (EPA)^17^. Comparable assessments are currently available for neither 1-methylnaphthalene nor the dimethylnaphthalenes. Acetophenone, which was also identified in the handbag, can lead to strong signs of intoxication according to the BfR, with an indoor air concentration of 80 ppm^6^.

Another matter of concern are the measured concentrations of PAHs in this product. According to the BfR, PAHs are relatively similar in their carcinogenic potential but their carcinogenic potency varies: The carcinogenic potency of naphthalene, for example, is 1000 times lower than the potency of benzo[a]pyrene^18^. In the investigated sample, the offensive smell raised the concern that the product might be detrimental to the health of consumers. However, the odour-active naphthalene, even if a compound of concern on its own, was not the only critical substance and other odourless compounds were found that are potentially hazardous. This demonstrates that odour may only provide a hint that a product is contaminated with substances that are not supposed to be there. Or vice versa, an odourless product may still contain critical substances, and lack of smell does not exclude their presence. In view of this it needs to be mentioned that a new European regulation is in power since December 2015, which prohibits the sale of toys that contain more than 0.5 mg/kg of benzo[a]pyrene, benzo[e]pyrene, benzo[a]anthracene, chrysene, benzo[b]fluoranthene, benzo[j]fluoranthene, benzo[k]fluoranthene or dibenzo[a,h]anthracene^19^. The measured content of crysene exceeds this limit, which would make the product not marketable in the European Union if it was introduced to the market now. However, the sample was purchased prior to that date, proving the necessity of such regulations. On the other hand, it is also interesting to note that no legally binding limits have been defined for several hazardous compounds such as naphthalene or most of the PAHs identified in the handbag, yet.

Our investigation showed, however, that there are much more potential compounds to be addressed in future studies, both with regards to their potential occurrence in other products, their concentration and the degree and dynamics of their emission. Only then it will be possible to evaluate potential physiological-toxicological aspects related to such smells. In view of this, it is important to consider the fact that these smells may not necessarily be associated with any “classical” toxicological harm, but that odour exposure may negatively impact humans by other mechanisms. Common physiological-toxicological considerations do rarely take into account perceptual and related physiological and psychosomatic effects, which are directly linked to the intermediate perception of smell. Moreover, it needs to be considered that children may be impacted even to a higher extent than adults as previous studies showed that children are olfactorily more sensitive than adults^20, 21^.

## Conclusions

In the present study, potent odorants, partially with offensive smells, in a fancy dress accessory for children were investigated. Thereby, more than 30 odorants belonging to a variety of chemical substance groups were successfully identified in the sample. It could be shown that the smell of the handbag correlated mostly with the smell of naphthalene and its derivatives. While several of the odorants found in the fancy dress accessory were identified in plastics and/or toys before, there were also substances identified that have not been reported as odorants in plastics yet. These are (*E,E*)-octa-2,4-dienal, (*E*)-oct-2-enal, 3-methylisoquinoline and raspberry ketone. Furthermore naphthalene, its methyl- and some dimethyl derivatives were found in plastics before, but their contribution to off-odours of plastics has not been discussed previously.

Amongst the identified odorous contaminations were also potentially hazardous substances such as naphthalene and acetophenone. However, to evaluate if the sample’s smell might pose a risk to children’s health further investigation of relevant exposure scenarios of these substances is required.

In addition to the odorous compounds, screening analysis for PAHs showed that other substances of concern were also present in the sample. However, out of the 15 PAHs identified in the sample only naphthalene was olfactorily active.

Accordingly, this study shows that the unusual smell of a product may be a hint for unwanted compounds; the final proof, however, can be only the analytical and physiological evaluation of such products and their constituents. Our study lays the foundation for future more targeted investigations and demonstrates the urgent need for intensified research in this field. The study also implies the need for stronger regulations on the chemical content of children’s products, such as limits for a broader range of specified PAHs, as well as regulations addressing the sensory properties of toys.

## Material and Methods

### Description of the sample

A handbag shaped like a witches’ caldron that was sold as an accessory for a children’s costume was tested in this study. The sample was (as declared on the packaging) made in China and obtained from an online supplier located in Germany. Upon arrival, the sample was kept in the original plastic packaging, wrapped in several layers of aluminium foil and stored at −80 °C until extraction. The sample was made from a black rubber-like material, which was identified as polyurethane.

### Chemicals

The following chemicals were obtained from the suppliers given in parenthesis:

(3S,5R,8S)-5-isopropenyl-3,8-dimethyl-3,4,5,6,7,8-hexahydro-1(2H)-azulenone (rotundone) (Symrise, Holzminden, Germany); (*E*)-non-2-enal ≥ 97%, (*E*)-oct-2-enal ≥ 94%, (*E,E*)-nona-2,4-dienal ≥ 85%, (*Z*)-non-2-enal, 1,3-benzothiazole (benzothiazole) ≥ 96%, 1,4-dimethylnaphthalene ≥ 95%, 1-methylnaphthalene ≥ 95%, 2,3-dimethylnaphthalene ≥ 97%, 2,6-diethylnaphthalene ≥ 97%, 2,6-dimethylnaphthalene ≥ 99%, 2,7-dimethylnaphthalene ≥ 99%, 2-methylbutanoic acid ≥ 98%, 3-hydroxy-4,5-dimethyl-5(2H)furanone (sotolone) ≥ 97%, 3-methylbutanoic acid ≥ 99%, 3-methylisoquinoline ≥ 98%, 4-(4-hydroxyphenyl)-2-butanone (raspberry ketone) ≥ 99%, 4-methylphenol (p-cresol) ≥ 99%, dodecanoic acid ≥ 98%, hexanal ≥ 98%, oct-1-en-3-one ≥ 50%, octa-2,4-dienal, predominantly trans, trans ≥ 95%, octanal ≥ 99%, phenylacetic acid ≥ 99% (Sigma-Aldrich, Steinheim, Germany); 1,2-dimethylnaphthalene ≥ 98%, 1,5-dimethylnaphthalene ≥ 99%, 1,6-dimethylnaphthalene ≥ 99%, 1,7-dimethylnaphthalene ≥ 97%, 2,3,5-trimethylnaphthalene ≥ 95%, hex-1-en-3-one ≥ 90%, vanillin ≥ 99% (abcr GmbH & Co. KG, Karlsruhe, Germany); (*E*,*E*)-deca-2,4-dienal ≥ 85%, butanoic acid ≥ 99,5%, naphthalene ≥ 99,7%, nonanal ≥ 95% (Fluka, Steinheim, Germany); 1,3-dimethylnaphthalene ≥ 96%, 1,8-dimethylnaphthalene ≥ 98% (ARCOS Organics, Geel, Belgium); 3-ethylphenol ≥ 98% (Riedel-de-Haen, Seelze, Germany); acetophenone ≥ 98% (SAFC, Steinheim, Germany); trans-4,5-epoxy-(*E*)-dec-2-enal ≥ 97% (AromaLab, Planegg, Germany); γ-octalactone (EGA Chemie, Steinheim, Germany); dichloromethane, distilled, sodium sulphate, anhydrous (Th. Geyer GmbH & Co. KG, Renningen, Germany); PAH-Mix 100 μg/ml in toluene: naphthalene, acenaphthylene, acenaphthene, fluorene, phenanthrene, anthracene, fluoranthene, pyrene, benzo[a]anthracene, chrysene, benzo[b]fluoranthene, benzo[k]fluoranthene, benzo[j]fluoranthene*, benzo[e]pyrene*, benzo[a]pyrene, dibenzo[a,h]anthracene, benzo[g,h,i]perylene, indeno[1,2,3-cd]pyrene (Neochema GmbH, Bodenheim, Germany); perdeuterated internal standard All-in-one 16 EPA priority PAHs 100 μg/ml in toluene, (Chiron AS, Norway); petroleum ether, picograde, toluene, picograde (Promochem, Wesel, Germany).

*no EPA-PAH’s

The (*E*,*Z*)-nona-2,4-dienal standard was isolated from an isomeric mixture.

### Solvent extraction of the sample

To extract the odorants from the plastic matrix, about one third of the handbag, including the inner layer, (10 grams in total) was cut into small pieces approx. 1 cm × 1 cm each. The sample and 200 ml of dichloromethane were put into an iodine determination flask and the solution was stirred for 30 minutes at room temperature.

Afterwards the solution was filtered and a Solvent Assisted Flavor Evaporation (SAFE) according to Engel *et al*.^22^ was performed to remove non-volatile compounds from the extract. For the distillation a water bath temperature of 50 °C was chosen and the SAFE-apparatus was kept at 55 °C to avoid condensation in the course of the distillation process. The distillate was dried over anhydrous sodium sulphate and finally concentrated to a total volume of 100 µl at 50 °C by means of Vigreux distillation and microdistillation according to Bemelmans^23^.

### Gas chromatography-olfactometry (GC-O)

Gas chromatographic separation was performed using a helium GC (Trace GC Ultra, Thermo Fisher Scientific, Dreieich, Germany) using the following capillaries: DB-FFAP (J & W Scientific 30 m × 0.32 mm fused silica capillary, free fatty acid phase FFAP, 0.25 µm; Agilent Technologies, Waldbronn, Germany) and DB-5 (J & W Scientific, 30 m × 0.32 mm fused silica capillary DB-5 0,25 µm; Agilent Technologies, Waldbronn, Germany).

The samples were applied to the GC system at 40 °C using the cool-on-column-technique. After 2 min, the temperature of the GC was raised at 8 °C/min to 230 °C and held for 10 min using the capillary DB-FFAP; for the capillary DB-5 the temperature was raised at the same rate to 240 °C and held for 5 min. The flow rate of the helium carrier gas was 2.2 ml/min. At the end of the capillary the eluent was split into a sniffing port and a flame ionization detector (FID), or alternatively a mass spectrometer (MS), using two deactivated, uncoated fused silica capillaries (50 cm × 0.2 mm). The FID and the sniffing port were held at 270 °C and 250 °C, respectively. The GC-O analyses were performed by five sensory trained panellists.

MS analyses were performed with a DSQ-II-system (Thermo Fisher Scientific, Dreieich, Germany) after gas chromatographic separation using the capillaries described above. Mass spectra in the electron impact (MS/EI) mode were generated at 70 eV ionization energy. The m/z range was 35 to 249.

For each odorant recorded by the panellists and each corresponding reference substance the linear retention indices (Ri) were calculated as described by Van den Dool and Kratz^24^.

### Two-dimensional GC-MS/O analysis

For the 2D-GC-MS/O analysis two systems were used with two methods, each optimized for the corresponding system.

System 1 consisted of two helium CP 3800 GCs (Varian, Darmstadt, Germany) in combination with a Saturn 2200 MS (Varian, Darmstadt, Germany); in system 2 two helium Agilent 7890A GCs (Agilent Technologies, Waldbronn, Germany) and an Agilent 220 Ion Trap GC-MS mass spectrometer (Agilent Technologies, Waldbronn, Germany) were used. In both systems, analytes were separated on a capillary DB-FFAP in the first oven and on a capillary DB-5 in the second oven. The specifications of the capillary columns were similar to those that were used for GC-O.

In both systems the samples were applied at 40 °C using the cool-on-column-technique. In system 1 the temperature of the first oven was raised 2 min after the injection at 8 °C/min to 230 °C and held for 2 min. In the second oven the temperature was raised at the same rate to 250 °C and held for 1 min. The flow rate of the helium carrier gas was 7.9 ml/min. At the end of each capillary, the effluent was split into a sniffing port and a FID or alternatively a MS, using two deactivated, uncoated fused silica capillaries (100 cm × 0.20 mm). The FID was held at 240 °C; for the sniffing-port a temperature of 290 °C was chosen. Mass spectra in the electron impact (MS/EI) mode were generated at 70 eV ionization energy. The m/z range was 35 to 399.

For system 2 the following parameters were used: The temperature of 40 °C of the first oven was held for 2 min, then raised at 8 °C/min to 240 °C and the final temperature was held for 5 min. In the second oven the temperature was raised at a rate of 10 °C/min and the final temperature of 240 °C was held for 5 min. The flow rate of the helium carrier gas was 7.8 ml/min. As in system 1, the effluent was split into a sniffing port and a FID or alternatively a MS at the end of each capillary, using two deactivated, uncoated fused silica capillaries (100 cm × 0.20 mm). The FID was held at 250 °C; for the sniffing-port a temperature of 300 °C was chosen. Mass spectra in the electron impact (MS/EI) mode were generated at 70 eV ionization energy. The m/z range was 35 to 249.

### Identification of odorants

Odorants were identified by comparison of the odour qualities, the retention indices on both capillaries DB-5 and DB-FFAP and, whenever possible, the obtained mass spectral data to those of reference compounds.

### Aroma extract dilution analysis (AEDA)

FD-factors were determined by AEDA^25^ to identify the most important odorants of the product’s smell. The following dilution series was used: The original extract (prepared as described in “solvent extraction of the sample”) was diluted stepwise with dichloromethane (1 + 2) to obtain seven solutions in total (FD 1 to 729). GC-O was then performed on aliquots of 2 µl of the extracts corresponding to FD 9 to 729 using capillaries DB-FFAP and DB-5.

### Sensory evaluation

#### Panellists

Panellists were trained volunteers from the Fraunhofer IVV Institute (Freising, Germany), exhibiting no known illness at the time of examination and with normal olfactory function. The panel consisted of five female panellists, aged 25 to 53. Prior to participation in the experiment panellists were tested for their olfactory functions during weekly training sessions with selected suprathreshold aroma solutions.

#### Descriptive analysis

Sensory analysis was based on a consensus profile analysis as described in the original industry standard DIN 10967-2, including our own in-house modification of the protocol with regards to the data evaluation and the used scale as will be detailed in the follwing. To obtain the odour profile, panellists were asked to describe the smell of the sample individually based on their orthonasal evaluation. Afterwards, common odour attributes were collected and rated on a scale from 0 (no perception) to 10 (strong perception) by all panellists. Furthermore, the panellists were asked to rate the overall intensity of the smell of the sample on a scale from 0 (no perception) to 10 (strong perception). Mean numerical values of all ratings were then calculated and plotted as a spiderweb diagram.

### Identification of the sample’s material using attenuated total reflectance spectroscopy (ATR-spectroscopy)

The identification of the material of the fancy dress accessory was carried out by ATR-FTIR-Spectroscopy on a Thermo Fisher Nicolet 5700 instrument. This infrared spectroscopy is used in case of samples which are not transparent. For analysis the sample and the crystal were pressed together, so infrared radiation can interact between these two materials. Certain infrared waves, which are reflected on the crystal’s surface, are diminished during this process, depending on the chemical composition of the sample. The FTIR-technique first records an interferogram of the reflected waves, which then is data-processed by Fourier transformation, and so turned into an infrared spectrum. The measurement took place between the wave numbers 4000 and 400 cm^−1^, corresponding to the mid-infrared spectrum. Identification of the sample’s material was carried out by comparison of the sample’s spectrum with reference spectra from the database belonging to the analysis-program OMNIC 7.3 using the following libraries: Aldrich Condensed Phase Sample Library, Georgia State Crime Lab Sample Library, HR Aldrich Polymers, HR Aldrich Solvents, HR Hummel Polymer and Additives, HR Polymer Additives and Plasticizers, Hummel Polymer Sample Library, Organics by Raman Sample Library, Sigma Biological Sample Library, Sprouse Polymer Additives and Sprouse Polymers by ATR/Transmission.

### GC-MS screening analysis for PAHs

For extraction of the PAHs from the sample’s plastic matrix, about 500 mg of the handbag were cut into small pieces of max. 3 cm × 3 cm each. The sample and 19.9 ml of toluene and 100 µl of an internal standard containing 16 perdeuterated EPA-PAHs (5 µg/ml) were added to an iodine determination flask. Subsequently the solution was ultrasonized for 60 minutes in a water-bath at 60 °C. After cooling down the solution to room temperature, a cleaning step with Florisil (Bond Elut FL, 1 G, 6 ml; Agilent) was performed. Florisil was conditioned with 5 ml petroleum ether and 3 ml toluene, before 2 ml of the extract was added. After the sample extract passed the Florisil material, another 5 ml toluene were added to elute the rest of the sample. Subsequently, the volume of the sample was reduced to 1000 µl by purging the solvent with a nitrogen stream at room temperature.

The GC-MS analysis of each extract was carried out on a GC-MS Instrument Shimadzu QP 2010 SE, equipped with a Zebron ZB-50 (50% Phenyl, 50% Dimethylpolysiloxane), 30 m × 0.25 mm ID × 0.25 μm capillary, in the electron impact mode. A sample volume of 1 µl was injected using the splitless mode. The oven temperature was started at 100 °C. After 2 min the temperature was increased with 5 °C/min to 240 °C. Subsequently, the temperature was increased with 2 °C/min up to 305 °C, where it was held constant for 10 min. Total runtime was 55 min.

### Ethics Statement

The study was conducted in agreement with the Declaration of Helsinki. The study (registration number 180_16B) was approved by the Ethical Committee of the Medical Faculty, Friedrich-Alexander Universität Erlangen-Nürnberg. Informed consent was obtained from all subjects participating in the study.
